# A High-Similarity Modeling Method for Low-Porosity Porous Material and Its Application in Bearing Cage Self-Lubrication Simulation

**DOI:** 10.3390/ma14185449

**Published:** 2021-09-21

**Authors:** Jiannan Sun, Ke Yan, Yongsheng Zhu, Jun Hong

**Affiliations:** Key Laboratory of Education Ministry for Modern Design and Rotor-Bearing System, Xi’an Jiaotong University, Xi’an 710049, China; sun392802414@stu.xjtu.edu.cn (J.S.); yszhu@mail.xjtu.edu.cn (Y.Z.); jhong@mail.xjtu.edu.cn (J.H.)

**Keywords:** porous oil-bearing cage, median filter, quartet structure generation set, slice method, pore structure model, simulation

## Abstract

The porous oil-containing cage achieves the storage, spillage, and suction of lubricating oil by its micro-pore structure, thus ensuring the self-lubricating performance of the bearing. Carrying out fast and accurate modeling of the cage microscopic pore structure is the key to the analysis of the self-lubricating mechanism of bearings. In response to the issues where current modeling methods of porous materials have a low similarity of pore distribution, morphology, structure, and size characteristics, and the transition of pore surfaces is sharp, this paper proposed a modeling method of a highly similar micro-pore structure based on the idea of median filtering, the quartet structure generation set (QSGS), and the slice method. By extracting and analyzing the pore characteristics of the porous model and comparing them with the experimental results of CT scanning, the advantages of the modeling method in terms of morphology and pore connectivity were verified. Finally, by carrying out simulation analysis of the centrifugal force of oil splashing and capillary oil absorption on the constructed model by combining the parameters of porous structures such as porosity and tortuosity, the advantages of the modeling method in the construction of the porous model and multi-physical field analysis were further verified.

## 1. Introduction

Owing to their excellent self-lubricating properties, oil-containing bearings are widely used in aerospace, microelectromechanical systems, deep-sea exploration, and so on. The self-lubrication of oil-containing bearings is achieved by the oil flow in and out of the porous media. As a kind of oil-containing bearing, bearings with a porous cage realize its self-lubrication through the oil flow circulation inside the micron and submicron pores of the porous cage. As the rotational speed rises, the lubricating oil stored in the porous cage continuously seeps out under the joint effect of centrifugal force, thermal effect, and capillary force to play a lubricating role on the friction surface. When the speed of the cage decreases, the lubricant on the cage surface is drawn back into the porous matrix under the capillary force of the micro-porous channel. In this way, bearing self-lubrication is realized. The pore structure of the porous cage is a key factor affecting the internal fluid properties, which further affects the bearing lubrication characteristics. Therefore, the study of the flow characteristics and flow mechanism inside the porous structure of the cage is a prerequisite for the study of its seepage process and self-lubrication performance.

At present, the lubrication performance of cages is mainly studied by various experimental means. For example, the Lanzhou Institute of Chemical Physics prepared porous polyimide (PI) materials with different pore parameters by the cold-pressing sintering technique and studied their performance of oil storage and tribological behavior [1]. In addition, the Henan University of Science and Technology [2], Nanjing University [3], Inha University in South Korea [4], and NASA in the United States [5] have also conducted relevant experimental studies on this topic. The above studies focused on the lubrication characteristics of the material by observing and summarizing the macroscopic patterns of the experiments. Unfortunately, it is difficult to directly obtain the flow process inside porous materials by experimental means, which leads to the inability to explain the lubrication mechanism. The pore structure has a significant impact on the flow characteristics and lubrication performance of the oil inside the bearing cage. Unfortunately, the pore structure inside the cage has a micron, or even submicron, scale and is highly complex, random, and disordered, making the set-up of the simulation model and the flow behavior analysis very difficult. Therefore, the key to investigating the self-lubrication mechanism of porous materials lies in the numerical modeling of the microscopic pore structure and the flow analysis of the internal pores, especially for porous oil-containing cage bearings, whose pore structure is characterized by tortuous, complex, low porosity, and small average pore size. The establishment of a highly restored numerical model similar to the real pore structure has become a prerequisite for the analysis of the lubrication mechanism of the cage.

Scholars have proposed various modeling methods for the microscopic pore structure of porous materials. In general, modeling methods for porous materials can be broadly classified into digital core methods and pore network models. The pore network model is an abstract and simplified model for the complex pore internal structure inside porous materials, which can be regarded as a combination of simple geometric shapes and features. Its basic principle is to divide the pore structure into some combinations of units with different functions. Among them, a throat is used to characterize the narrow pore space, a pore body is used to represent the larger pore space, and a regular geometry is used to characterize the pore body and throat. The structure of the model constructed by this method is simple and can visually represent the parameters of the pore structure (such as pore size and coordination number) inside the material. The disadvantages of the pore network model are also obvious: it differs greatly from the real pore structure, resulting in a low degree of similarity. In addition, it cannot quantitatively analyze the degree of irregularity in porous materials. When the pore morphology of porous materials is complex, the accuracy of the model is greatly reduced, which can easily cause large deviations. In summary, the pore network model provides a means to extract the structural parameters of pores and to study the flow behavior of pore-scale fluids but lacks an effective method to quantitatively describe the degree of irregularity of porous materials. The common modeling methods for pore network models include the maximal ball algorithm (MB), the watershed algorithm (WA), and the medial axis algorithm. In 2019, Todor et al. used the maximal ball algorithm and the watershed algorithm to extract the pore network model [6]. In addition, Wang combined the maximal ball algorithm and the medial axis algorithm to extract the pore network model from 2D SEM images in 2020 [7]. In addition, the ball-and-stick model structure employed in the PNM approach is also applied in the field of molecular dynamics analysis, thus simplifying and analyzing the particle simulation for convenience. Similar approaches were utilized in the literature [8,9] for the modeling and simulation analysis of a nanoparticle in oligomeric poly(methyl methacrylate) and triblock Janus particles, respectively. Given the complexity of the pore structure with submicronic scale inside the porous cage material, the existing pore network model is not suitable for the modeling of porous cages. However, as the model can easily extract the structural parameters of the porous material, the pore network model can be used as an auxiliary means during pore structure characteristic analysis.

Digital cores are digital models used to characterize the microstructure of porous materials. The current method of modeling the digital cores can be divided into two main categories: image reconstruction and numerical reconstruction. The method of image reconstruction mainly acquires a series of two-dimensional images of materials by imaging devices such as scanning electron microscopy and CT tomography and then constructs three-dimensional models using reconstruction methods such as the slice method. In 1997, Zhu built up a three-dimensional microstructure reconstruction system using stereo technology of a scanning electron microscope [10]. This method can visually reproduce the material structure, but it is time-consuming and requires high equipment requirements. The principle of the numerical reconstruction method is to construct an image model using mathematical algorithms based on the statistical information of the material image. It mainly includes the simulated annealing method, Gaussian random field method, multi-point statistics (MPS) method, the random seed method, the quartet structure generation set (QSGS) method, etc. For example, Hidajat et al. combined the Gaussian field method and simulated annealing method to construct a porous material model, which extremely improved the modeling efficiency [11]. However, the Gaussian field method and the simulated annealing method only perform some calculations of flow simulation directly on the reconstructed image and do not extract the available porous material model [12]. Hajizadeh et al. used the MPS method to achieve the reconstruction of the 3D pore space from 2D training images [13], which avoided the need for 3D images in the traditional MPS method and thus improved the computational speed. Renmin et al. used the QSGS method to construct a two-dimensional microstructure model of clay and investigated the groundwater percolation in the clay [14]. The construction process of this method has similarity with the generation process of porous materials, but the models constructed by this method are mostly two-dimensional images, and the original three-dimensional models generated are point data. It is difficult to use this method to build solid models, and it cannot be directly applied to the numerical analysis of multi-physics field coupling. Recently, in the pore-scale modeling of porous oil-containing cages, Yin et al. proposed a modeling method for the 2D/3D structure of porous cages based on the random seed method. A pore structure model, which has a certain degree of similarity in terms of quantitative indicators (porosity and pore size) and qualitative indicators (pore morphology and pore distribution), was obtained [15]. However, due to the Boolean operation between seeds, cusps appear at the pore edges of the 3D model constructed by Yin et al., which lead to distortion of the pore structure. More importantly, the cusps in the model make the meshing process difficult, resulting in the inability to perform simulation calculations in software. Due to this, the simulation of the single-phase flow inside porous materials mainly uses the lattice Boltzmann (LBM) approach and mainly focuses on the acquisition of absolute permeability [16]. 

In summary, the current modeling method is mainly based on a two-dimensional model, and the three-dimensional model has a low model similarity and easy distortion, which is difficult to apply to multi-physics field-coupled finite element simulation software. The analysis of the 3D model is mainly based on the LBM method, and many models and flow field types cannot be analyzed and calculated. For example, the color-model is one common LB model for simulating multiphase flow in porous media, while it is limited to density-matched fluids, and numerical stability issues arise for large viscosity ratios and/or small capillary numbers [17]. Besides, Fakhari et al. proposed a conservative phase-field lattice-Boltzmann model for ternary fluids, which has shown promising results. However, it reduces the fidelity of the simulations compared with the FD-based approach [18].

It can be seen that it is necessary to improve the digital core-based modeling method for better meshing and simulation analysis of 3D models. This is the key to researching porous materials. To this end, in this study, according to the manufacturing process of porous materials, a modeling method that combines the QSGS method and the slice method was proposed. The pore boundary structure by the median filtering method was optimized and the pore surface was generated and interpolated by Avizo software using the slicing method. In this way, the tips and distortions of the random seed method were avoided, which made the constructed pore structure closer to the actual morphology of porous materials. According to this method, the meshing of the 3D model of the pore structure and the simulation analysis within the finite element software were realized. On this basis, the pore network model was used to extract multiple sets of parameters for the porous material model, and the generated 3D model was compared and analyzed with the prepared specimens. Finally, the simulation study of the oil dumping by centrifugal force and the capillary oil absorption of porous materials was carried out according to the constructed porous material model. The simulation results were verified with experimental data and then analyzed in combination with the relevant pore structure parameters. The model meshing processing, calculation efficiency, and simulation results reflect the advantages of the modeling method by this paper.

## 2. Modeling Principle

**Figure 1:**
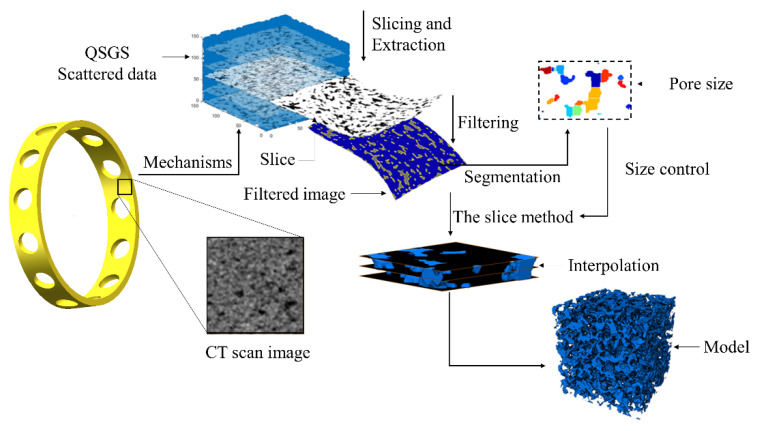
Modeling idea.

In this paper, the QSGS method was employed to realize the random growth of the pore structure inside the porous material and to generate the scattered points of the pore structure. Then, based on the generated point data, the slice graphs of the pore structure were extracted, resulting in a set of continuous slices. Subsequently, the median filtering algorithm was used to filter the stray points within the slice graph and optimize the boundary of the pore structure within the slice. Then, the watershed algorithm was employed to segment the processed slice maps, calculate the average pore size of the pore structure, and adjust the size of the model space based on the comparison of the calculated average pore size with the expected value. Finally, via Avizo software, the processed slice graphs were combined to generate the intermediate surface and construct the corresponding 3D pore structure model. The modeling principle is shown in Figure 1.

## 3. Modeling Process

**Figure 2:**
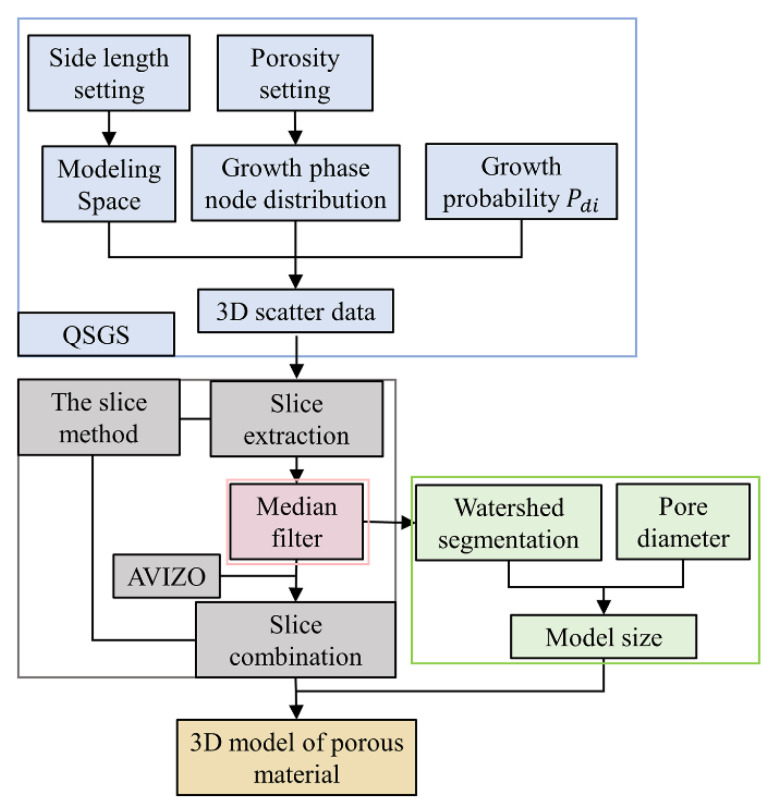
Modeling process.

Based on the above modeling idea, slices were extracted from the output of the QSGS method, processed by image filtering and segmentation, and reused as the input of the slice method. Subsequently, MATLAB and Avizo 3D reconstruction software were used to construct the 3D solid model of the pore structure of porous materials. The modeling flow is shown in Figure 2.

### 3.1. The QSGS Method

In this paper, the bonding of solid particles during the preparation of porous materials was considered as the growth of the growth phase in the spatial range. Based on this, the quartet structure generation set (QSGS) method was used to generate the three-dimensional scatter data needed to construct a three-dimensional model of porous materials. The QSGS method was first proposed by Wang [19], which adjusts the growth process of particles of porous materials by introducing the concept of growth probability. Thus, a growth model for porous materials with pores and matrix solid particles was generated.

In this paper, the pore was considered as the nongrowth phase and the matrix solid-phase as the growth phase. The specific process is as follows.

(1) Set the dimensions (*lx*, *ly*, *lz*) and porosity n of the microscopic model, spread all the meshes within the set 3D porous model dimensions, and generate the growth phase nodes randomly, with each node representing a solid phase particle.
(2) Set the growth probability and growth direction of the growing phase nodes so that the solid particles grow randomly in the three-dimensional space. The growth directions include 6 principal directions (P_d1_–P_d6_) and 12 angular directions (P_d7_–P_d18_). Due to the isotropic nature of the porous material, set P_d1_–P_d6_ to be equal and P_d7_–P_d18_ to be equal.
(3) Iterate over the node coordinates of all growth phases, and assign a random number between 0 and 1 in each growth direction of each node. If this random number is less than the corresponding probability P_di_, add a growth phase node along the corresponding direction.
(4) Count the number of the nodes of the growth phase, and calculate the porosity reached during growth to see if the porosity defined is reached; if not, repeat step (3).
(5) The growth mechanism of the QSGS method leads to a lower growth probability of the grid edge than that of the internal growth phase. Therefore, remove the model boundary for a specific length range from the original pore model.

**Figure 3:**
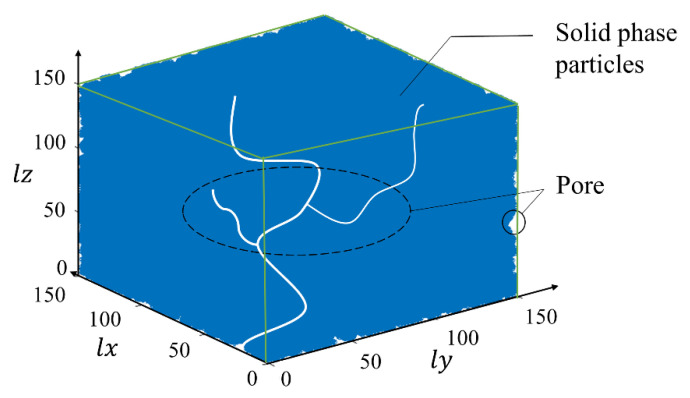
Scatter plot of pore model.

Based on the above process, the program for the QSGS method was written in MATLAB, and 3D scattering maps representing the pores and the matrix solid particles were generated. The scatter diagram of the generated pore model is shown in Figure 3. The data points in the diagram represent the matrix solid particles of the material, and space not covered by the points in the cubic modeling space constitutes the pore structure of the porous material model.

### 3.2. Slice Extraction and Median Filtering

**Figure 4:**
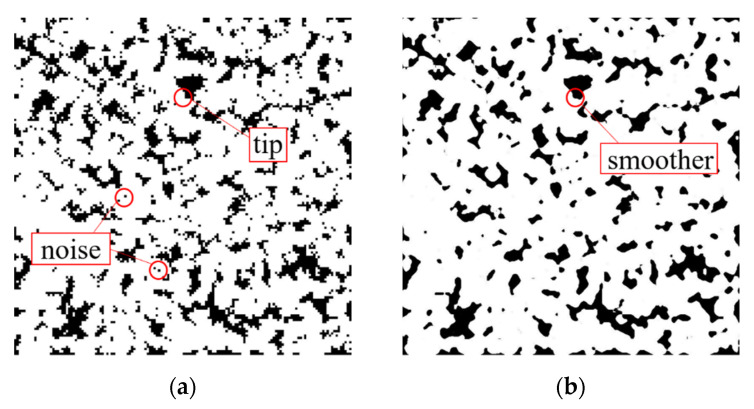
Slice extraction and median filtering: (**a**) Original slice map; (**b**) Median filtered image.

According to the generated scatter data graphs, these scatter data can be considered as a stack of planar data images along the Z-axis. Based on this idea, the pore structure data were extracted and the slices for subsequent 3D modeling were reorganized using the slice method. The slice method is a popular method used in 3D reconstruction software. The principle is to generate a sufficient number of slices of continuous 3D structural cross-sections by grinding, cutting, and image acquisition of experimental specimens. After that, the intermediate surfaces between the slices are generated by sequential superposition and image processing techniques, finally constructing the resulting 3D structure images. This method requires high accuracy for the relevant experimental equipment. In this paper, the X and Y coordinates of all growth phases in each layer were extracted along the Z-axis, and the binary slice maps were generated based on the obtained coordinate data. The continuity of the pore and matrix solid phases within the generated set of slice maps can be ensured on a three-dimensional scale because the scatter maps are randomly grown in space from the growth phase. Besides, as the object of slicing is not an entity but a binary data point map, the requirement for experimental equipment is avoided. The obtained slice image is shown in Figure 4a. Furthermore, as can be seen from Figure 4a, many growth phase nodes failed to grow sufficiently during the generation of the continuous pore structure, resulting in many noise points inside the images obtained by slice extraction. Moreover, the pore slice image in MATLAB was made by stacking particles of the growth phase, and each growth phase was a pixel point. Due to the limitation of the computational speed of the computer, the size of the modeled space set was small and the pixel points constituting the pore space were relatively large. These pixel points were connected, resulting in a stepped pore structure boundary.

Based on the microscopic morphology of porous materials and considering the simplification of calculations in subsequent simulations, tips should be avoided as much as possible when generating meshes for porous models. In this paper, the nodes of the growth phase that fail to grow sufficiently in the image were treated as speckle noise, they were filtered by the median filtering method, and the pore boundaries were smoothed.

Median filtering is a nonlinear signal processing technique developed based on the statistical theory of order, which was first proposed by Turky in 1971. This method is widely used because of its simple algorithm structure, good noise-reduction effect, and better protection of the edge details of the image from blurring. The steps of median filtering are as follows: To begin with, divide an odd-length sampling window in a digital image or sequence. Then, sort the samples in the window by grayscale value. Next, generate the monotonically increasing or monotonically decreasing data sequence. Finally, replace the value of the window centroid with the grayscale value of the middle sample of the sequence. In this way, the filtering of noise is achieved. The median filtering of two-dimensional digital images is specified as follows.

Set the window as a two-dimensional template of size n × n and the grayscale value of the original image as *f*(*x*,*y*), and define the filter window as *A* and the grayscale value of the median filtered image as *g*(*x*,*y*); then, the median-filtered output of the filter window is

(1) g(x,y)=Med{f(x±l,y±m),(l,m)∈A},

The window used for filtering varies in its applicable shape depending on the processing object. In this paper, a square window was used considering the shape characteristics of the pore structure. The effect of noise reduction using median filtering is shown in Figure 4b.

### 3.3. Watershed Segmentation and Pore Size Control

Taking the filtered slices of the porous material model as samples, the watershed segmentation algorithm was used for pore size segmentation to obtain the average pore size of the holes in the slice images, which facilitated the subsequent resizing of the model to obtain the desired pore size.

The watershed segmentation algorithm is a region-based method whose concept was proposed by Digabel et al. based on mathematical morphology and further developed by Vincent and Soille [20]. In this paper, the watershed algorithm was used for the segmentation of binary images. Its principle is based on the elevation gradient of natural topography in topography. When rain falls from different locations on the surface, it converges at the local minimum surface of the terrain, forming a connected area called a “catchment basin,” and the junction line of the basin not covered by rain is called a “watershed.” Based on this idea, the watershed algorithm converts the grayscale image into a gradient map and then extracts the boundaries and catchment basins to achieve the segmentation of the image [21].

The watershed segmentation algorithm can be described as:

(1) The image is processed by the gradient operation to obtain the corresponding gradient map, and let *h*(*x*,*y*) be the processed gradient image; then:

(2) h(x,y)=grad{f(x,y)},

where *grad* represents the function of gradient operation.

(2) Let *M_1_*, *M_2_*, … *M_R_* be the local minima of the gradient image, *C*(*M_i_*) be the set of coordinates adjacent to *M_i_*, and *T*(*n*) denote the set of coordinates with gradient *h* less than *n*. Take *C_n_*(*M_i_*) as the set of *T*(*n*) adjacent to the minima point *M_i_*, i.e.,

(3) $$C_{n}(M_{i})=C(M_{i})\cap T(n).$$

Take *C*[*n*] as the union of each *C_n_*(*M_i_*) in the image, representing the connected catchment area covered by rainwater in each minimal area (valley) in the gradient map.

(4) $$C[n]=\overset{R}{\underset{i=1}{\cap }}C_{n}(M_{i}).$$

In this way, the process of covering rainwater from low to high in the valley is realized, thus achieving the segmentation process of the image.

**Figure 5:**
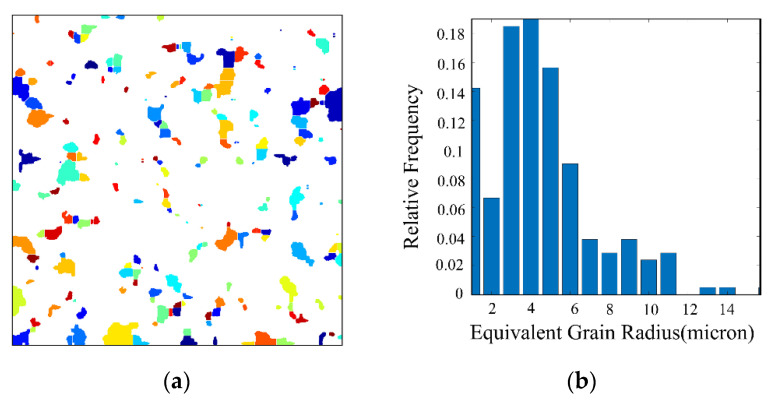
Watershed segmentation: (**a**) Pore size segmentation; (**b**) Equivalent pore size distribution.

The segmented image is shown in Figure 5.

The area of each segmented region was counted, and the average pore size was calculated by considering them as circular pores:

(5) r=mean{∑i=1nResolution*Si/π},

where *n* is the total number of pores segmented in the slice image, Re*solution* is the spatial resolution in microns/pixel, and *S_i_* is the area of the *ith* pore.

In this paper, the obtained average pore size was compared with the preset value and, thus, the model size that should be set for the subsequent 3D reconstruction was calculated. In this way, we can adjust the average pore size of the model of porous materials. Subsequently, the pixel size of the image was resized using the imresize function to ensure that the pixel of the subsequent 3D model was cubic. This process caused a loss of sharpness in the 2D image, but benefitted the slice method to interpolate the model surface in the subsequent 3D reconstruction, thus optimizing the surface and making it smoother.

### 3.4. Model Reconstruction and Meshing

**Figure 6:**
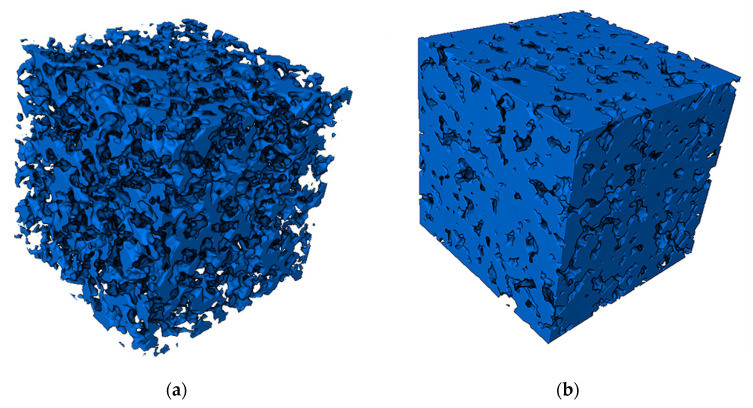
Solid model of porous material: (**a**) Pore structure (watershed); (**b**) Matrix structure (solid).

Finally, the processed set of pore model slices was imported into Avizo software to generate the intermediate surfaces by the slice method to construct the porous material model. Afterward, the model surface was interpolated to construct the desired porous material model. The pore structure and material matrix structure of the obtained 3D solid model are shown in Figure 6.

**Figure 7:**
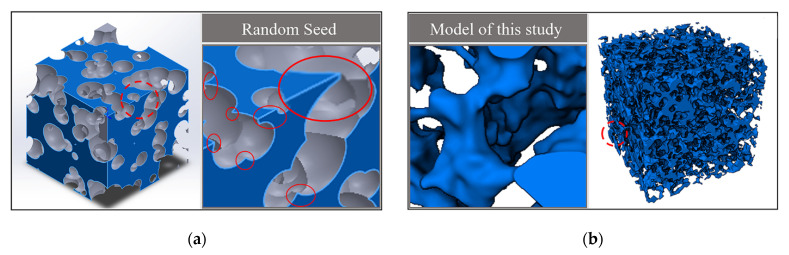
Comparison of modeling methods: (**a**) The random seed model; (**b**) Modeling model of this study.

In this paper, the model established in this study was compared with the 3D model constructed by Yin et al. using the random seed method, and the details of both models were enlarged for analysis and comparison, as shown in Figure 7.

As shown in Figure 7, the traditional random seed modeling method distributed the spherical pores randomly in the modeling space and constructed the pore model by the Boolean operation between balls. As can be seen from the enlarged details, the pore structure of the random seed model differed from the actual morphology of the porous material, and the intersection of the balls led to the existence of sharp angles in the model, which did not match the actual structure of the porous material and was not conducive to the model meshing and simulation analysis, so the simulation of the 3D model of the porous material was not successfully carried out based on this model. In contrast, the present modeling method adopted the median filtering algorithm and the slice method to optimize the intermediate surface, which successfully avoided the spiking phenomenon caused by the Boolean operation between the spherical pore models. From the figure, we can see that the morphology of the model was more similar to the actual material, the surface was smoother, and the transition of the pores was more natural, which was conducive to the 3D meshing and simulation analysis of the solid model.

**Figure 8:**
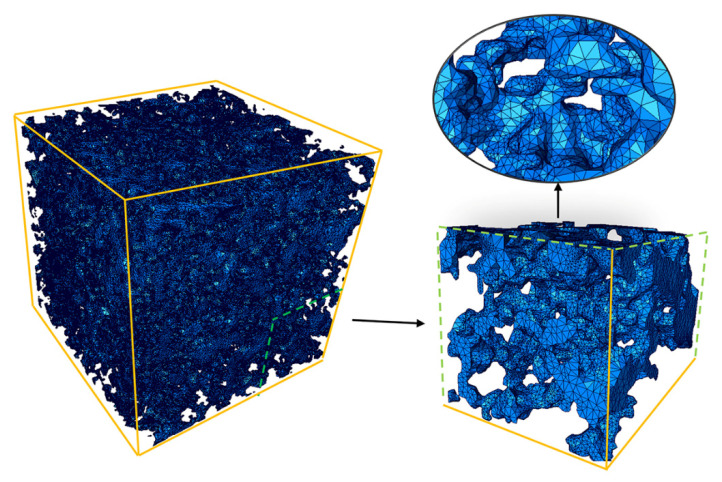
3D solid mesh model.

The mesh model of the three-dimensional model of porous material was successfully constructed by this method, as shown in Figure 8. The mesh division was more uniform, and the similarity with the actual shape was higher, which was conducive to the subsequent import of software for simulation analysis.

## 4. Structural Analysis of Porous Material Model

### 4.1. Experimental Verification

**Figure 9:**
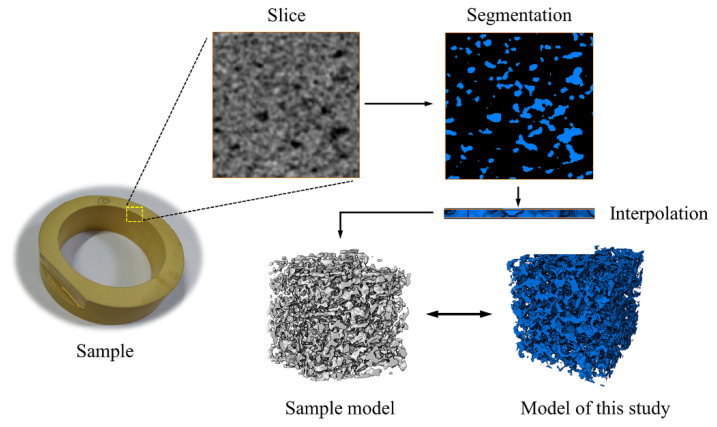
The slice and model of the specimen.

To verify the accuracy of the modeling method in this paper, the MPPI02-type porous polyimide cage material by Luoyang Bearing Research Institute Co., Ltd. was selected. The sample was scanned on an X-CT scanning equipment (GE Nanotom M) to obtain the slicing figures of the cage material and the watershed method was used to extract the pore structure. Then, the slice method was used to construct the intermediate surface by Avizo software and thus generated the 3D structural model of the specimen material. Finally, the model of the specimen was compared with the model constructed in this paper. As shown in Figure 9, the solid part of the model characterized the pore structure, and the space inside the cubic model represented the material matrix. We can see that the models constructed by the modeling method in this study had different geometries of pores and zigzag pore structures, which have a high similarity to the microscopic morphology of porous materials. In Section 4.2, we further compare the model of our study with the real material from the aspect of structural parameters, such as pore size distribution and sphericity, which verified the accuracy of the modeling method in this paper.

### 4.2. Analysis of Structural Parameters of Porous Materials

In the modeling method used in this study, AVIZO software was used for modeling and analysis, which can easily extract the pore structure parameters of the model. We obtained the pore diameter, coordination number, sphericity, fractal dimension, and tortuosity of the model, which are beneficial to the study of the structure and lubrication performance of the porous oil-containing cage. The extracted pore structure parameters of the model are as follows.

#### 4.2.1. Pore Size Distribution

**Figure 10:**
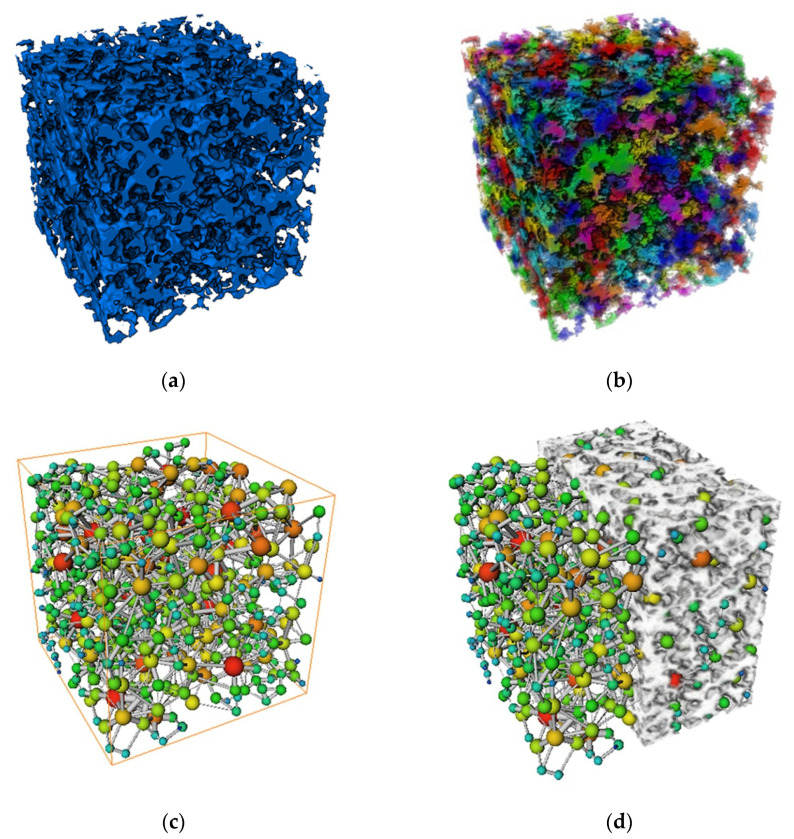
Construction of the PNM model: (**a**) Pore structure model; (**b**) Segmentation model; (**c**) PNM model; (**d**) PNM-material matrix.

The pore network (PNM) model was extracted from the porous material model based on the maximal ball algorithm. The principle of the maximal ball algorithm is to use spheres of different radii to fill the pore channels, and then characterize the pore bodies by spheres and the pore throats by cylinders, thus constituting a ball-and-stick model to represent the pore structure of porous materials [22]. Figure 10 shows the extraction process of the PNM model. First, we modeled the pore structure of porous materials (Figure 10a), and we then segmented the model (Figure 10b) to generate the corresponding spheres and rods, which constituted the PNM model (Figure 10c). From Figure 10d, it can be seen that the PNM model generated by the maximal ball method was embedded between the porous material matrix (off-white model), indicating that the PNM model matched well with the pores of the porous material.

**Figure 11:**
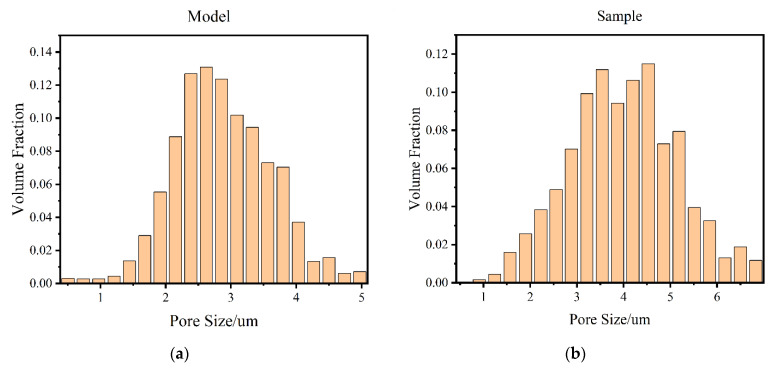
Pore size distribution: (**a**) Pore size distribution of the model in this study; (**b**) Pore size distribution of prepared samples.

The pore diameters were collected according to the generated PNM model, and the histogram of the pore diameter distribution of the solid model was obtained and compared with the prepared specimens for verification, as shown in Figure 11. It can be found that the model used in this study and the prepared samples had a high similarity in terms of pore size distribution, and both of them roughly conformed to the normal distribution. The accuracy of the modeling method in this study was further verified.

#### 4.2.2. Coordination Number

**Figure 12:**
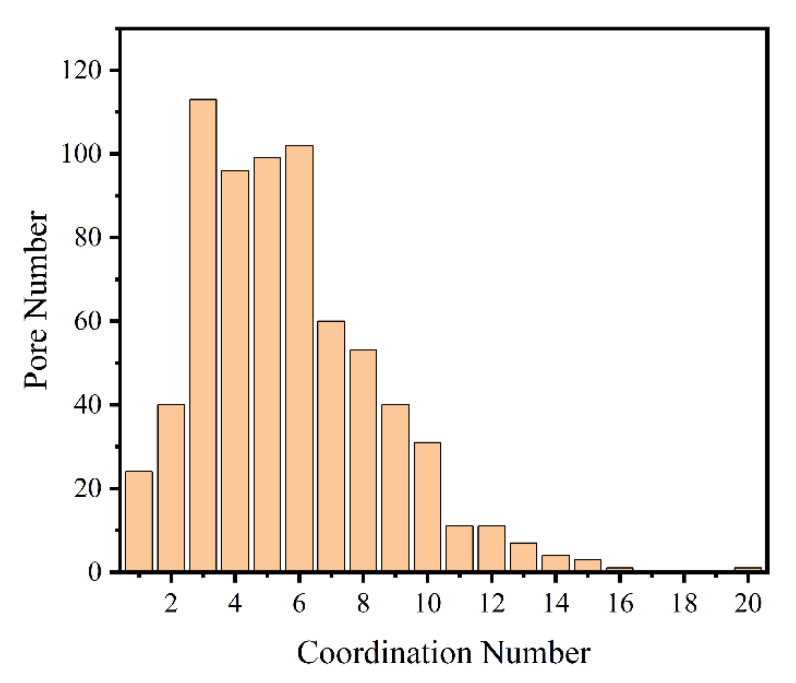
Distribution of the pore numbers with different coordination numbers.

The coordination number refers to the number of throats connected by a central pore in porous materials. It is a parameter describing the degree of connectivity between pores. Based on the model established in this paper, the PNM method was further used to simplify the model in this paper, and the number of pores with different coordination numbers was counted as a percentage of the total number of pores, as shown in Figure 12. In addition, based on the statistical data, the average coordination number of the model can be calculated to characterize the connectivity performance of the porous material.

#### 4.2.3. Sphericity

Sphericity is a measure of the degree of sphericity of an object. In this paper, sphericity was used to characterize the regularity of the segmented pore structure. The equation to calculate the sphericity is as follows.

(6) $$\psi =\frac{\pi^{1/3}{\left(6V\right)}^{2/3}}{A},$$

where *V* is the volume of pores and *A* is the surface area.

**Figure 13:**
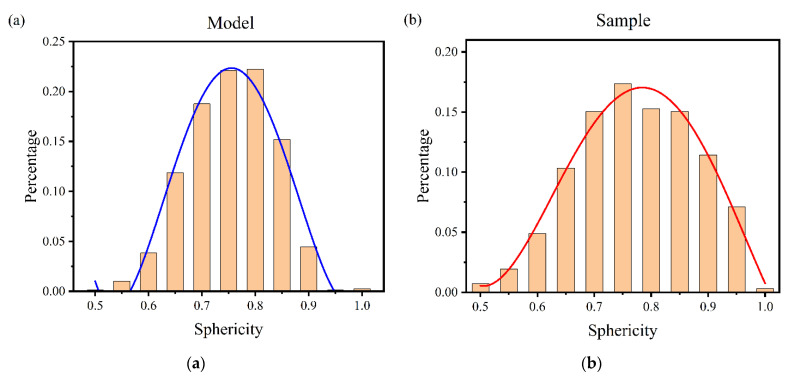
Sphericity vs. percentage of pores. (**a**) Percentage of the number of pores with different sphericity in the model of this paper; (**b**) Percentage of the number of pores with different sphericity in the sample.

Based on the porosity of the specimens, a porous material model with a porosity of 0.17 was constructed and the sphericity of the segmented pores in the model was extracted. The ratio of the number of pores with different sphericity to the total number of pores was calculated, and the fitted curves were plotted for comparison with specimens of the cage material, as shown in Figure 13. We can find that the sphericity of the pore structure model had approximately the same statistical law as the specimen, which further indicates that the model constructed by this modeling method was in good agreement with the actual specimen in terms of morphology.

#### 4.2.4. Fractal Dimension

The fractal geometry theory proposed by Mandelbrot is of great importance for describing the irregularity of the pore structure of porous materials. The fractal dimension is an important indicator to measure the irregularity of complex forms. Numerous studies have shown that the pore structure of porous materials has fractal properties. In this study, the fractal dimension of the pore structure of porous materials can be easily extracted using Avizo software.

To clarify the relationship between fractal dimension and porosity, five sets of porous material models with a porosity of 0.235 were constructed, and the fractal dimension of each model was extracted, as shown in Table 1. We can see that the difference of fractal dimension among the models was small when the porosity did not change much, so the fractal dimension can be used to characterize the porosity of porous materials.

**Figure 14:**
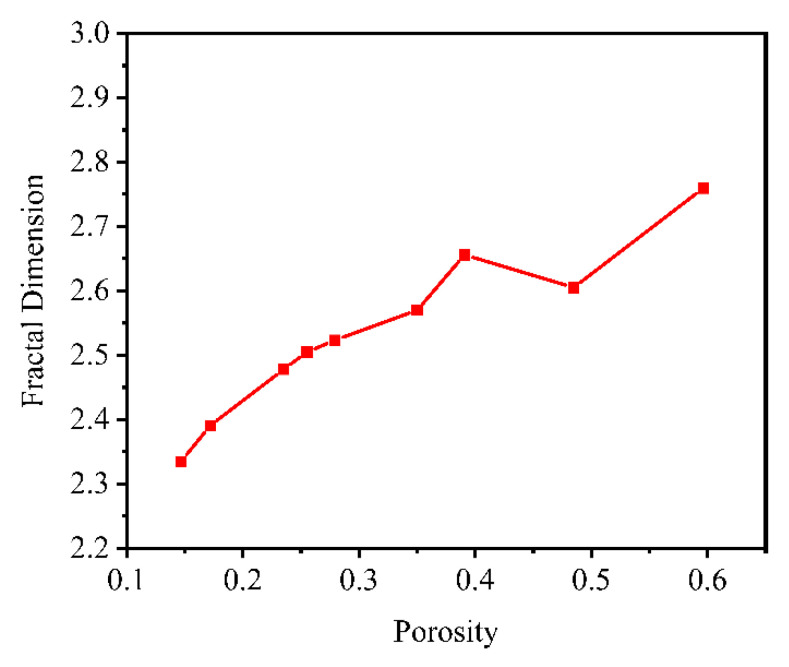
Porosity vs. fractal dimension.

Then, in this paper, nine groups of models with different porosities were established to extract the fractal dimension, and the results are shown in Figure 14. It can be seen that the fractal dimension of porous materials was roughly positively correlated with the porosity, which is consistent with the conclusions of the literature [23] on the relationship between porosity and the fractal dimension of rocks.

#### 4.2.5. Tortuosity

The internal structure of the porous cage is complex, and the fluid shows a tortuous flow inside the cage. In this study, the tortuosity of the model was extracted and applied to subsequent simulations to investigate the relationship between tortuosity and the lubrication performance of porous oil-containing cages. 

**Figure 15:**
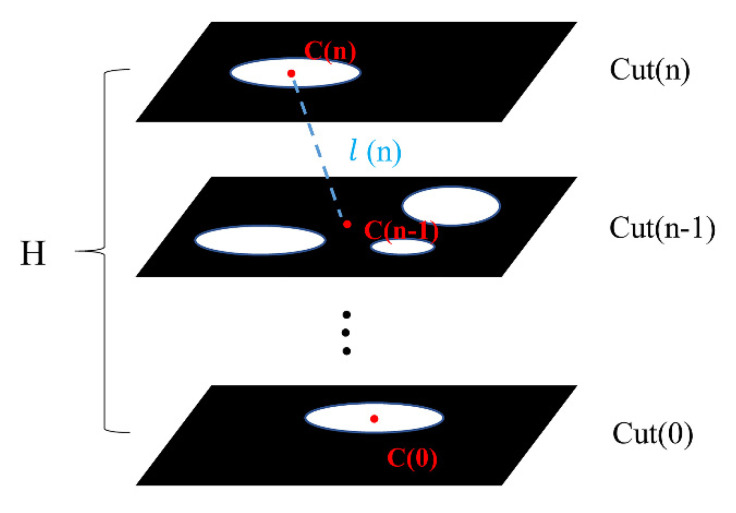
The centroid path tortuosity.

In this paper, the tortuosity of the porous material was characterized by the centroid path tortuosity. A path formed by the centroids on each plane along the z-axis of a binary 3D image was used as the curved length, as shown in Figure 15. The tortuosity is calculated as follows:

(7) τ=L/H,

where *L* represents the curved path and *H* represents the distance along the *Z*-axis at each end of the path. The length of *L* can be expressed as the accumulation of the distance between the centroids of each adjacent slice:

(8) $$L=\sum_{i=1}^{n}l(i),$$

### 4.3. Control of Porosity and Pore Size

Presently, the porosity and pore size of the models constructed based on image reconstruction and pore network models are related to the specimen material and are difficult to control. The model structure generated by methods such as the random seed method is more regular. In addition, the Boolean operation between pores causes a loss of pore volume, leading to a higher deviation of porosity. In this paper, the watershed segmentation algorithm was used to segment the slices of the model, which can effectively control the parameters such as porosity and pore size of the model. This is beneficial to study the effect of porosity on the lubrication performance of porous cages.

The connected pore structure (Avizo-connectivity) of the solid model was extracted and its effective porosity was calculated; then, the porosity was compared with the porosity set by MATLAB and the total porosity of the solid model (Avizo). As shown in Table 2, it can be seen that the total porosity and effective porosity of the solid model constructed in this study were relatively close to the porosity set by MATLAB, indicating that the model established in this paper has good accuracy and connectivity.

This modeling method used the watershed algorithm to segment the graph and calculated the model size based on the preset pore size, thus realizing the control of the average pore size. The present modeling method still had some defects: the segmentation algorithm used during the model construction was different from the segmentation method (MB algorithm) used in the subsequent extraction of pore size, leading to some differences in the calculation results. However, the pore size ratio remained unchanged, which facilitated the qualitative analysis of the average pore size of porous materials, whereas the quantitative analysis is subject to further study.

## 5. Simulation

By building a porous material model, a multi-physics simulation of fluid flow can be performed to study the fluid properties inside the cage. At present, it was difficult for the porous material model constructed by the existing numerical reconstruction method to generate a solid mesh model, and the simulation was difficult. The modeling method in this paper, based on the idea of combining filtering and slicing, can effectively construct a usable three-dimensional mesh model of porous materials with a smoother model surface, which is conducive to the finite element simulation of the fluid inside the pores. Taking the 7008C bearing porous oil-containing cage as the background, COMSOL Multiphysics software was used to study the centrifugal force of the oil splashing simulation and the capillary suction simulation of the cage according to the established model, and to analyze the influence of pore structure parameters on the self-lubricating performance of the porous cage. A simulation study of the established pore model of the porous cage was conducted by referring to the simulation setup of Yin et al. [24] and the case on the official website of Comsol. In addition, porous materials can also be applied to the field of concrete; for example, in the literature [25,26], the authors analyzed the mechanical properties of concrete. The input parameter selection and analysis ideas in the above work have also been used for reference in this article.

### 5.1. Centrifugal Force Simulation

#### 5.1.1. Creeping Flow Equation and Boundary Conditions

In this paper, COMSOL Multiphysics^®^ Version 5.6 was used for the simulation analysis. The single-phase flow fluid flow interfaces are based on the Navier–Stokes equation. As the pore scale in the porous material is micron scale, the flow velocity of oil in the pore is very slow, so the fluid flow in the pore can be regarded as creeping flow, neglecting the inertia term in the Navier–Stokes equation, and the equation becomes the Stokes equation:

(9) $$\rho \frac{\partial u}{\partial t}=\nabla \cdot [-pI+\tau ]+F,$$

where *ρ* is the density, *u* is the velocity vector, *p* is the pressure, *τ* is the viscous stress tensor, [*τ−p*I] represents the viscous force in the tangential direction, and F is the volume force vector.

**Figure 16:**
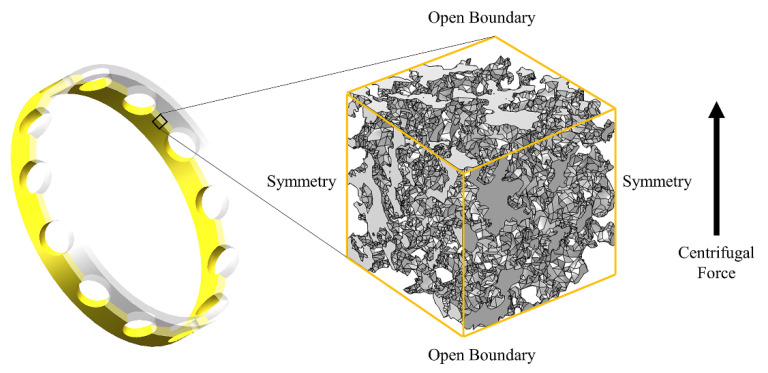
Boundary conditions for centrifugal force simulation.

In this study, the boundary conditions were set as shown in Figure 16, the upper and lower boundaries of the model were set as open boundaries, and the remaining faces were set as symmetric faces. Besides, in the centrifugal force simulation, the volume force was set as the centrifugal force according to the cage speed (2000 rpm in this paper), the material density (polyimide material was used), and the cage radius, and the porous model size was 50 µm. The centrifugal force was calculated as follows:

(10) $$F_{\mathrm{RCF}}=\rho (z+R)\omega^{2},$$

where F_RCF_ is the centrifugal force, *R* is the cage’s inner diameter, and, as the 7008C bearing cage was used in this study, *R* was selected as 0.028 m.

#### 5.1.2. Simulation Results

**Figure 17:**
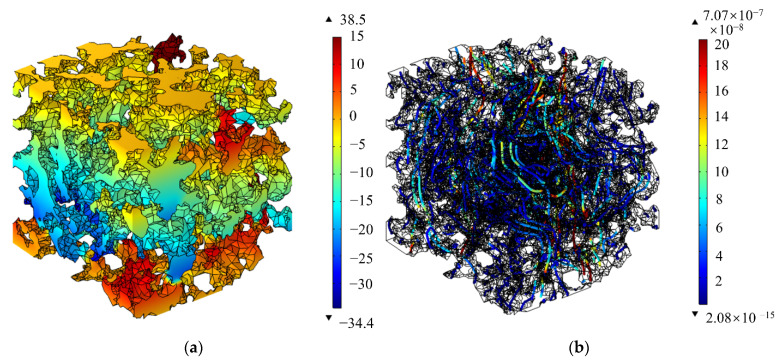
Pressure and velocity streamlines: (**a**) Pressure distribution; (**b**) Velocity streamlines distribution.

**Figure 18:**
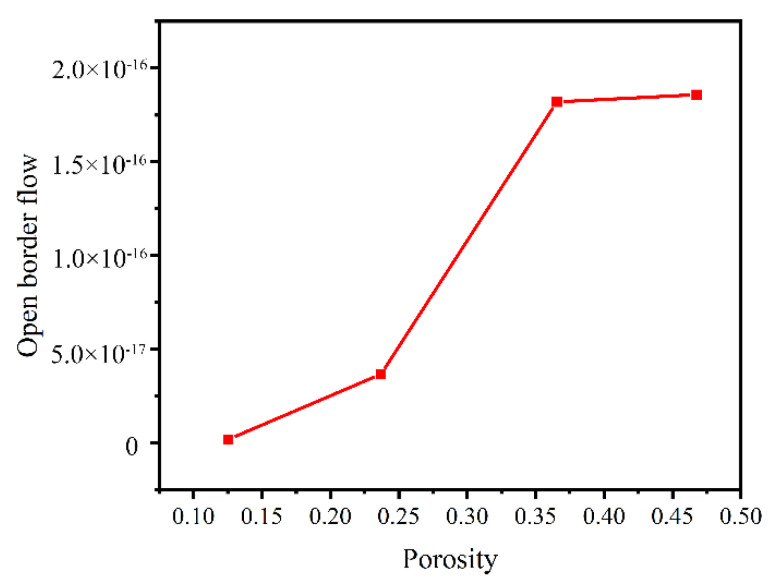
Oil output from porous materials.

1. Simulation Process
  The transient solver was selected to study the oil flow out of the porous material under the action of centrifugal force. The input of the simulation is shown in Table 3.
  The oil pressure and velocity streamlines in porous materials were obtained, as shown in Figure 17.
2. As can be seen in Figure 17, the distribution and structure of the pores in a porous material had a significant effect on the local pressure and flow rate of the fluid. Within the porous material, the pressure at the blind end of the pore growing in the direction of the centrifugal force was more drastic and the oil pressure is higher. The closed pores growing in the opposite direction of the centrifugal force had a lower oil pressure because it was difficult to replenish the fluid. Besides, it can be seen that the flow velocity was faster in the orifice with less curvature of the material structure and smooth flow lines. Effect of porosity on oil output;

Models with different porosities were selected, and the outlet flow at the open boundary of the model under the action of centrifugal force was calculated. The parameters of the calculation models 1–4 are shown in Table 4, and the results are shown in Table 5. According to the results, the effect of porosity on the oil output of the cage under centrifugal action was analyzed, as shown in Figure 18. From the figure, it can be seen that the outlet flow of the porous material model gradually increased with the porosity. It can be further concluded that with the increase in porosity of the porous oil-containing cage material, the oil output of the cage increased. This phenomenon also coincides with the experimental findings of Wang et al. [3] and proves the correctness of the method. In addition, it is worth emphasizing that only porosity and average pore size were controlled in this paper, while the oil output of porous materials was also related to many structural parameters; for example, the distribution of pore size also affected the oil output of porous materials under the same porosity. The control of pore size distribution was difficult to achieve at present, and it is hoped that future models can be further improved in this respect.

### 5.2. Capillary Force Simulation

#### 5.2.1. Level Set Equations, Wetted Wall, and Boundary Conditions

In this study, the interfaces were based on the Navier–Stokes equations in incompressible form. By using the level set tracking interface, the following equations were added:

(11) $$\frac{\partial \phi }{\partial t}+u\cdot \nabla \phi =\gamma \nabla \cdot \left(\varepsilon \nabla \phi -\phi (1-\phi )\frac{\nabla \phi }{\left|\nabla \phi \right|}\right),$$

where *ϕ* is the fluid volume fraction, *γ* is the reinitialization parameter, and *ε* is the interface thickness control parameter.

The fluid density function is defined as:

(12) $$\rho =\rho_{1}+(\rho_{2}-\rho_{1})\phi .$$

The dynamic viscosity function is defined as:

(13) $$\mu =\mu_{1}+(\mu_{2}-\mu_{1})\phi .$$

where *ρ*_1_ and *ρ*_2_ represent the density of fluid 1 (air) and fluid 2 (oil), respectively, and *μ*_1_ and *μ*_2_ represent the dynamic viscosity of fluid 1 and fluid 2, respectively.

In this paper, the wetted wall boundary condition was used to realize the movement of the fluid–fluid interface along the wall of the pore structure.

For creeping flow, this boundary condition enforces the no-penetration condition u·n_wall_ = 0, where n_wall_ is the unit normal to the wall, and adds a tangential stress:

(14) $$K_{nt}=-\frac{\mu }{\beta }u,$$

where *K_nt_* = *K_n_* − (*K_n_*·n_wall_)n_wall_, *K_n_* = *K*n_wall_, and *K* is the viscous stress tensor. *β* is the slip length. For numerical calculations, a suitable choice is *β* = *h*, where *h* is the mesh element size. The extrapolated tangential velocity component is 0 at a distance *β* outside the wall.

The wetted wall boundary condition also adds the following boundary force to enforce the contact angle:

(15) $$F_{\theta }=\sigma \delta (n_{\mathrm{wall}}\cdot n\;-\;\cos\theta_{w})n,$$

where the contact angle *θ_w_* is defined directly in this paper. *σ* is the surface tensions coefficient and n is the unit normal to the interface. The *δ*-function is approximated by a smooth function according to

(16) δ=6|∇ϕ||ϕ(1−ϕ)|.

**Figure 19:**
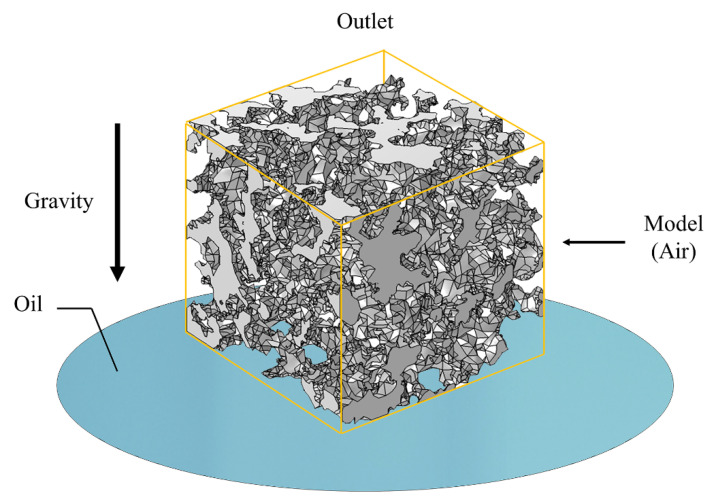
Boundary conditions for capillary force simulation.

A coupled transient analysis of the level set and creeping flow physical field was used, ignoring the inertia term. The wetted wall module was added to the multi-physical field coupling interface and the contact angle was set. The time was set to 0.002 s, the time step was 0.02 × 10^−3^ s, and an oil pool was added below the pore model. Then, the oil absorption of the porous material model under gravity and capillary force was simulated. The boundary conditions of the simulation model are shown in Figure 19.

#### 5.2.2. Simulation Results

**Figure 20:**
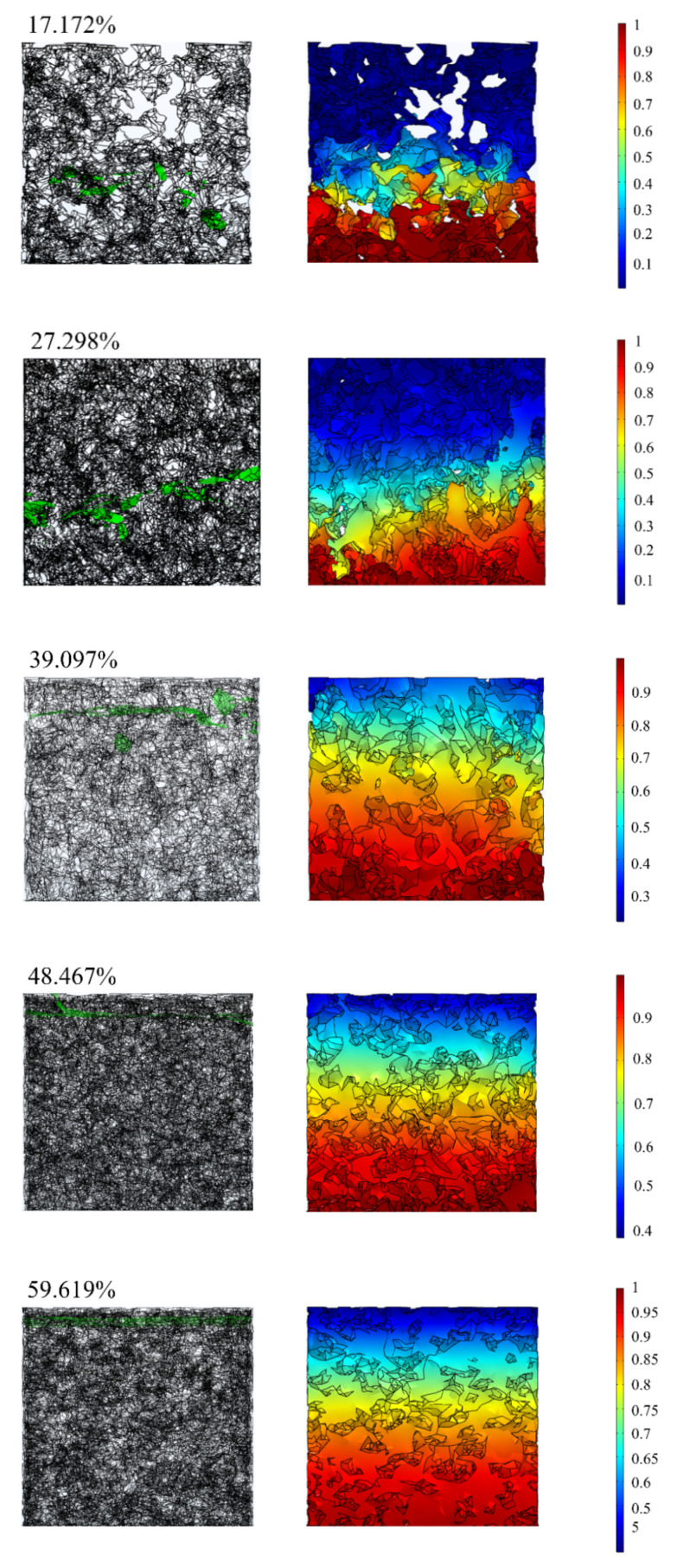
Oil level (**left**) and oil volume fraction (**right**).

Based on the modeling method adopted in this study, the pore structure models of porous materials with different porosities were constructed, the average pore size was set to 1 µm in MATLAB, and the porosities of the models were 17.172%, 27.298%, 39.097%, 48.467%, and 59.619% respectively. Based on these models, the oil absorption of porous materials under capillary action was simulated. The oil level and volume fraction of oil inside the porous material model are shown in Figure 20. The input of the simulation is shown in Table 6.

The 3D model established by the traditional modeling method has various problems, such as sharp points and a nonsolid model, which make it difficult to be applied to the finite element method. In addition, the models developed by traditional porous material modeling methods are mainly used to calculate permeability and rarely deal with other aspects, such as capillary effects. From the simulation results, it can be seen that the modeling method in this paper is more applicable to the finite element simulation, which can be used more explicitly for analysis under different working conditions and is more practical. This also proves the feasibility of the modeling method in this paper in performing finite element simulation. 

Besides, porous materials have a high degree of disorder. Among the structural parameters about the real porous materials, porosity, pore size and its distribution, tortuosity, fractal dimension, and pore sphericity interact with each other, and all of them have a high randomness, which will have an impact on the computational results. The focus of this paper was to realize model modeling with a high degree of similarity to real porous materials. The model of our study has a high consistency with some parameters of the actual material, such as the porosity and pore size. However, it must be acknowledged that the calculation results of building different models with the same porosity and average pore size still have a large difference. This law is similar to the real porous materials. In addition, the goal of our future study is to further control other structural parameters of porous materials, such as tortuosity.

## 6. Conclusions

This paper took the porous oil-containing cage as the research background. The real porous material structure is highly disordered, and its modeling methods are mostly based on simplified models, with nonsolid models, low similarity, and easy distortion, which make it difficult to be applied to multi-physics field-coupled finite element simulation software. The analysis of the 3D model is mainly based on the LBM method, and many models and flow field types cannot be analyzed and calculated. In response to the above problems, a study of modeling methods for 3D porous material models and the feasibility of the simulation was conducted. In this paper, a modeling method based on the combination of the QSGS method and the slice method was proposed, the model was compared with the actual porous material, and finally the simulation analysis was carried out to verify the feasibility of the modeling method.

(1) Considering the process of pore generation in porous materials, this study proposed a modeling method of a highly similar micro-pore structure based on the idea of median filtering and the combination of the quartet structure generation set (QSGS) method and the slice method. The pore structure scatter data of porous materials were constructed by the QSGS method. In addition, the median filtering method, watershed segmentation method, and the slice method were used to optimize the pore structure, control the pore size, and build the solid model, respectively. The model had a curved structure and smooth transition of pore structure, which had high similarity with porous materials. In addition, the original numerical modeling method was difficult for simulation software analysis, while the model constructed by the modeling method in this paper used the median filtering algorithm and interpolation to optimize the edges of the pore structure, avoiding the influence of tip and distortion on the mesh division, resulting in the constructed model being beneficial to meshing and finite element software simulation. Compared with the traditional modeling methods, the 3D solid model constructed by the method in this paper is more natural; moreover, the porosity and average pore size of the model can be effectively controlled.
(2) Based on the constructed porous material model, the pore structure parameters of the model, such as pore diameter, coordination number, sphericity, fractal dimension, and tortuosity, were extracted and analyzed to facilitate the study of the structure and lubrication performance of porous oil-containing cages. The pore size distribution and sphericity distribution of the model were compared with the experimental results of CT scanning, and the reasonableness of the model was verified.
(3) Based on the 3D model generated by the above modeling method, the simulation of oil splashing by centrifugal force and the simulation of capillary oil absorption for porous materials were performed. Combined with structural parameters such as porosity, the effect of pore structure on the self-lubricating performance of the porous oil-containing cage was analyzed. The feasibility of the modeling method proposed in this study was verified by comparing the simulation results with the experimental findings of others.

## Figures and Tables

**Table 1:** Fractal Dimension.

Porosity	Fractal Dimension
0.235056	2.47984
0.236298	2.47773
0.235618	2.48099
0.23559	2.47853
0.234892	2.47744

**Table 2:** Comparison of model porosity.

Model	Porosity
MATLAB	0.26
Avizo	0.2374
Avizo-connectivity	0.2356

**Table 3:** The input of the simulation.

Model Settings	Value
Model Size [um]	50 × 50 × 50
Porosity	27.298%
Cage speed [rpm]	2000
Output time [s]	Range (0, 0.025, 5)
Pore size (Matlab) [um]	1
Material	Polyimide
Physical Field	Creep flow
Temperature (K)	303

**Table 4:** Parameters of the calculation model.

Model	Porosity
1	12.545%
2	23.691%
3	36.541%
4	46.759%

**Table 5:** Oil output statistics.

Model	Oil Output [m^3^/s]
1	1.92 × 10^−18^
2	3.66 × 10^−17^
3	1.82 × 10^−16^
4	1.86 × 10^−16^

**Table 6:** The input of the simulation.

Model Settings	Value
Model Size [um]	50 × 50 × 50
Porosity	17.172%, 27.298%, 39.097%, 48.467%, 59.619%
Output time [s]	range (0, 0.02 × 10^−3^, 0.002)
Average Pore size (Matlab) [um]	1
Material	Polyimide
Physical Field	Creep flow, level set
Temperature [K]	293
Contact angle [°]	15
surface tension coefficient [N/m]	0.04
Dynamic viscosity [Pa·s]	0.799407 (oil), 1.81323 × 10^−5^ (air)
Density [kg/m^3^]	887.6808 (oil), 1.205 (air)

## Data Availability

The data presented in this study are available on request from the corresponding author. The data are not publicly available, due to the fact that they are also part of an ongoing study.
